# Epigenetic mechanisms underlying subtype heterogeneity and tumor recurrence in prostate cancer

**DOI:** 10.1038/s41467-023-36253-1

**Published:** 2023-02-02

**Authors:** Goutam Chakraborty, Kasmira Gupta, Natasha Kyprianou

**Affiliations:** 1 Departments of Urology, New York, NY USA; 2 Oncological Sciences, New York, NY USA; 3 Pathology & Cell Based Medicine, New York, NY USA; 4 Tisch Cancer Institute; Icahn School of Medicine at Mount Sinai, New York, NY USA

**Keywords:** Prostate cancer, Prostate cancer

## Abstract

Prostate cancer is a highly heterogeneous disease. Progression on androgen deprivation therapy (ADT) to castration-resistant (CRPC), or neuroendocrine prostate cancer (NEPC), is associated with poor patient survival. This comment highlights recent evidence on the epigenetic mechanisms underlying the emergence of lineage plasticity and neuroendocrine differentiation in treatment-resistant prostate tumors.

## The landscape of neuroendocrine prostate cancer

In the United States, over 90% of patients are initially diagnosed with prostate cancer when the disease is confined to the prostate or has begun to spread to the area just outside the gland^1^. The growth of hormone-sensitive prostate cancer (HSPC) is primarily driven by androgens (testosterone)^2^, and consequently, patients with recurrent prostate cancer after surgery (prostatectomy) or radiotherapy frequently undergo androgen deprivation therapy (ADT)^2^. However, for the majority of patients, prostate tumors ultimately become resistant to ADT and antiandrogens and resume growth despite diminished androgen levels, developing into metastatic castration-resistant prostate cancer (mCRPC), the lethal form of the disease^3–5^.

Given the genomic complexity and phenotypic heterogeneity that characterize prostate tumors, a single somatic or germline variant is unlikely prognostic. Various genomic changes are enriched in mCRPC compared to the primary diseases, such as loss of function mutations in the tumor-suppressor genes TP53, RB1, and PTEN, deficiency of DNA damage repair pathway, a gain of function mutations in oncogenes, and copy-number alterations^6–9^. However, despite significant advances in molecular genetics and genome analysis, our ability to distinguish lethal mCRPC from non-lethal indolent prostate cancer is still limited.

Neuroendocrine Prostate Cancer (NEPC) is a lethal subset of CRPC in which the tumor grows independently of the androgen receptor (AR) function/signaling and acquires a neuroendocrine phenotype^10,11^. A neuroendocrine phenotype is characterized by smaller, denser cells with compact nuclei and granules replete with neuroendocrine hormones^12^. Furthermore, the neuroendocrine phenotype is associated with the acquisition of cellular plasticity or epithelial–mesenchymal transition (EMT), in which a cell diverges from its well-differentiated epithelial phenotype, improving cell mobility and the ability to metastasize^13,14^. The neuroendocrine transition also allows for enhanced ability to secrete neurotransmitters that promote the aggressive behavior of cancer cells^15^. NEPC may arise de novo, but it is much less likely than NEPC caused by drug-induced inhibition of AR signaling, reinforcing tumor growth and resistance to AR inhibitors^10^. Neuroendocrine cells in prostate tumors are characterized by TP53 and Rb1 mutations, expression of neuroendocrine differentiation markers such as synaptophysin (SYP), neuron-specific enolase (NSE), neural cell adhesion molecule 1 (CD56), and absence of AR^16,17^.

Epigenetic regulation is a major contributor to cancer progression; consequently, epigenetic-targeted therapy is currently explored in hematological malignancies and solid tumors. Recent studies demonstrated that the epigenomic landscape of mCRPC has been remarkably reprogrammed compared to the indolent treatment naïve prostate cancer^18,19^. These epigenetic modifications reprogram the cistrome of various transcription factors and affect downstream gene expression, thus driving prostate tumor cells towards plasticity and neuroendocrine differentiation. Epigenetic plasticity including alteration of DNA methylation, nucleosome remodeling, and histone modification leads to deregulation of gene expression, contributing to disease progression^20^. Specifically epigenetic reprogramming is responsible for silencing tumor-suppressor genes, activating oncogenic drivers, and reprogramming the cistrome of key transcription factors (TFs) as the AR^20–22^. While clinical trials have been exploiting therapeutic strategies to target epigenetic modifiers such as EZH2^23^; the therapeutic impact of such strategies can be improved if applied at biomarker-defined points during the phenotypic transition between luminal epithelial and adaptive mesenchymal stem cell states.

## Molecular subtypes contributing to lineage plasticity

Defining the molecular evolution dictating the cellular heterogeneity during progression to NEPC in preclinical models can facilitate therapeutic targeting of tumor lineage plasticity^24^. On an epigenetic level, alteration of TFs binding, and chromatin modulators can affect gene expression, thus driving neuroendocrine subtype differentiation^18,20^. Cistrome reprogramming of transcription factors is the master regulator of an extensive gene network that affects NEPC progression^25^.

Characteristic examples of epigenetic regulators specifically expressed in NEPC cells include polycomb group proteins, heterochromatin protein, protein DEK, sucrose non-fermentable complex, and long non-coding RNAs^11^. Polycomb group proteins (PcG) form multiprotein Polycomb repressive complexes that play a major role in epigenetic gene silencing, cell growth, and cell differentiation^26^. PcG utilizes many mechanistic pathways, one being the modification of chromatin through the methylation of histone tails. However, the suppression of PcG is also found to lead to the progression of cancer^26^. Polycomb Repressor Complex 2 (PRC2, comprising EED and SUZ12) could transdifferentiate CSPC cells (LNCaP-AR) into NEPC and induce resistance to enzalutamide^27^. Recent clinical evidence supports that enzalutamide treatment in localized CSPC induces epigenomic plasticity^28^, by reprograming circadian rhythm regulation, which leads to the activation of cellular plasticity with the gain of NEPC-like features^28^. One may also consider the evidence that nitric oxide (NO) signaling acts as a driver of epigenetic reprogramming^29^, in the context of NO-signaling also inhibits the AR activity in prostate cancer cells^30^. This effect of NO-signaling on prostate tumor neuroendocrine differentiation consequential to impairing AR, provides a new insight into epigenetic reprogramming, begging further investigation. The epigenetic reprogramming dynamic can act as a driver, as well as a marker of ADT-induced cellular plasticity and neuroendocrine differentiation of indolent CSPC.

Neuroendocrine cancer cells (NECs) in prostate cancer patients present themselves as a small cell lung cancer (SCLC) like pathological phenotype, with diverse differentiation status of prostate cells. Chromatin landscapes in NEPCs can converge to present epigenetic similarities, and NE- tumors with overlapping profiles utilize a similar set of TFs. Recent studies have shown that NEPC phenotype in human cancer driven individually by two transcription factors, ASCL1, and NEUROD1 regulate distinct genes that contribute to neuronal functions and malignant behavior^31^. ASCL1, was recently identified to epigenetically remodel chromatin and stimulate neuronal stem cells, by regulating EZH2 activity and enhancing H3K27me3. ASCL1 can be induced by ADT in a subset of HSPC^32^. Targeting ASCL1 let to EZH2 phosphorylation on T311 via AMPK and a loss of H3K27me3 activation of luminal and AR target genes including KLK3. The effect of ASCL1 loss was not specific to H3K27me3 but rather universal and suggested a role of ASCL1 in methionine metabolism to support epigenetic reprogramming towards the emergence of a high plasticity neuroendocrine state^32^. ASCL1 is also an androgen-regulated gene and its loss abrogates antiandrogen-induced plasticity, suggesting that ASCL1 is required for treatment-induced plasticity^32^., implicating initiation of an ASCL1 clone by trans-differentiation from adenocarcinoma post antiandrogen treatment to drive NEUROD1 subclone^31^ and progression to NEPC. This further highlights the therapeutic value of targeting ASCL1 in early prostate cancer to impair the emergence of lineage plasticity and lethal disease. While in PDX models of NEPC ASCL1 and NEUROD1 are mutually exclusive, in patients, the existence of two distinct NEPC subtypes based on the expression of the neuronal transcription factors ASCL1 and NEUROD1 require larger datasets and longer clinical follow-up to define the subtype heterogeneity in NEPC^32^. For instance, heterogeneous subtypes that coexist within prostate tumors may differ in chromatin, histone, and DNA modifications such as methylation and acetylation patterns^33^. This heterogeneity often poses an obstacle to effective clinical treatment as distinct molecular subtypes within the same tumor are resistant to contrasting treatments.

A prior study showed that BRN2 a POU domain neuronal transcription factor, serves as a master regulator of NEPC progression^34^. Mechanistically BRN2 physically interacts with transcription factor SOX2 and they co-occupy the enhancer region of NEPC marker NSE and RFX4 in CRPC cells, to promote NE-differentiation from CRPC cells^34^. SOX2 is highly expressed in NEPC and promotes lineage plasticity of prostate cancer cells^16^. Importantly SOX2 regulates global epigenetic modulation via inducing Lysine-specific demethylase1 (LSD1) dependent hypomethylation of histone H3^35^. Therefore, the BRN2-SOX2 axis can reprogram the epigenetic landscape of prostate cancer cells towards the regulation of NE differentiation. Downregulation of the epigenetic remodeling factor REST also influences AR function and promotes NEPC progression by inducing multiple neuronal-associated genes such as BDNF, NTRK3, Syn1, and Grin2A^36,37^.

## Targeting epigenetic reprogramming in neuroendocrine prostate cancer

The DNA methylome distinguishes NEPC from CRPC. 5-methylcytosine (5mC) is the most abundant epigenetic marker of the vertebrate genome and essential for gene regulation^38^. Recent advancements in sequencing techniques, provide valuable tools for interrogating the epigenomic landscape of cancer cells and have enabled the mapping of 5mC, providing a genome-wide coverage of epigenetic changes^39^. Current whole genome-based epigenomic approaches include (i) Methylated DNA immunoprecipitation (MeDIP) uses an antibody specific to 5mC for immunoprecipitation followed by DNA sequencing,^40^; (ii) MethylCap methods employ a methyl-binding domain (MBD) protein to affinity-based capture of DNA fragmentation^41^; (iii) Restriction enzyme based methods take the advantage differential digestion property of methylation-sensitive restriction enzymes followed by sequencing (MRE-seq).^42^; (iv) Bisulfite method converts nonmethylated cytocines to uracils and introduce methylation-specific single nucleotide polymorphism (SNP), can provide single base resolution to investigate methylation-specific DNA sequences when couples with Sanger sequencing^43^^.^ In addition, chromatin immunoprecipitation (ChIP)-sequencing that utilizes the combination of ChIP with sequencing can identify the genome-wide binding sites of TFs^44^. Moreover, the Assay for Transposase-Accessible Chromatin using sequencing (ATAC-seq) is based on direct in vitro transposition of sequencing adaptors in native chromatin, which enables the genome-wide profiling of TF binding events and identification of lineage-selective transcriptional regulators^45^.

Phenotypic plasticity of prostate tumor cells, as defined by EMT to MET (mesenchymal to epithelial transition) interconversions, plays a critical role in the development of therapeutic resistance to ADT and antiandrogen therapy^46^. The functional interplay of chromatin remodeling and reprogramming AR-binding sites (AR-cistrome) has been associated with enzalutamide resistance in the epithelial-derived component of CSPC at the single-cell level. Taavitsainen and colleagues recently identified divergent chromatin reprogramming induced by enzalutamide exposure leading to therapeutic resistance and epithelial plasticity^45^. Moreover, using ATAC-seq and RNA-seq data from prostate cancer organoids, patient-derived xenografts, and cell lines, investigators have identified four different prostate cancer subtypes and predicted the key TF of each subtype^47^. This study identified two new AR-low/negative non-neuroendocrine subtypes of CRPC, driven by different master TFs, resulting in a distinct pathological subtype^47^. Open chromatin regions of cells contain promoter regions of actively transcribed genes, as well as non-coding regulatory sequences, contributing to the dynamic gene regulatory networks that drive cell state transitions. Single-cell chromatin landscape analysis (ATAC-seq indexing) of primary prostate tumors identified that high-grade tumors are enriched in transcription factor binding sites FOXA1, HOXB13, and CDX2, compared to indolent tumors^48^. Significantly, two genes encoding neuronal adhesion molecules (NRXN1 and NLG1) are highly accessible in advanced prostate cancer, and this epigenetic loss of heterogeneity results in neuroendocrine differentiation of prostate tumor cells^48^. Recent studies revealed that AR-targeted therapy induces transcriptional plasticity of intermediate cells towards the acquisition of NEPC, which is reversible in PTEN-deficient prostate cancer cells^49^; this evidence implicates a required presence by the intermediate cells to facilitate the transition of AR-high cellular subtypes to neuroendocrine phenotype.

As the impact of global epigenetic changes on therapeutic resistance and prostate tumor recurrence emerges, interrogating the chromatin landscape using ATAC-seq in a large cohort of prostate biopsy specimens (pre and post ADT) will provide a highly informative platform to study the contribution of epigenetic reprogramming to neuroendocrine phenotype and guide clinical-decision making regarding therapeutic interventions. Due to histopathological and genetic similarities between NEPC and SCLC, NEPC patients are often treated with systemic therapy regimens used for SCLC^50^. Platinum-based chemotherapy is the standard for treating NEPC; however, the responses are short-lived^50^. Based on the high expression of Aurora kinases (AURKA and AURKB) detected in NEPC tumors^51^, a phase II trial with alisertib (Aurora kinases A/AURKA specific inhibitor) was conducted for CRPC and NEPC patients. However, there was no significant improvement in overall survival, with disease progression in 88% of treated patients, leading to treatment discontinuation^52^. Thus, there is an urgent need to develop more durable therapeutic interventions for patients with NEPC. The epigenetic drugs currently approved for cancer therapy belong to two categories: targeting DNA methylation by DNMT inhibitors and histone acetylation by HDAC inhibitors^53^. Preclinical studies showed that targeting the epigenome in CRPC or NEPC with DNMT and HDAC inhibitors could lead to a potential therapeutic response^53,54^. Prior studies showed that therapeutic targeting of transcription and epigenetic regulator ONECUT2, which is also a driver of NEPC, with a small molecule inhibitor, significantly reduces mCRPC progression in preclinical mouse models^55,56^. Tyrosine kinase SRC regulates EZH2 expression in cancer cells and drives epigenetic reprogramming^57^. Combinatorial treatment of SRC inhibitors (dasatinib, bosutinib) with PARP-inhibitors (olaparib, talazoparib) or platinum-based chemotherapy remarkably reduces NEPC growth in preclinical GEM-derived model^58^. The recent emergence of proteolysis targeting chimeric (PROTAC) molecules has enabled targeted degradation of EZH2^59^ as well as several other undruggable proteins, including TF’s^60^. PROTAC-mediated degradation of the AR and AR variants ((ARV-110, ARV-471, and ARV-766) is currently being investigated in prostate cancer clinical trials^61,62^.

## Conclusions and future prospects

A significant subset (30%) of patients with CRPC progress to tumors exhibiting loss of their luminal identity and acquisition of neuroendocrine features. The cellular subtype heterogeneity results in therapeutic resistance and ultimately lethal disease. Here we discuss new insights into the epigenetic reprogramming of plasticity and neuroendocrine phenotype among cell subtypes, and the therapeutic significance in recurrent prostate cancer. Targeted degradation of NEPC-specific TFs and epigenetic regulators using PROTAC is a potential therapeutic strategy for treating lethal disease. Further exploitation of the epigenomic landscape of advanced prostate tumors will facilitate the development and implementation of novel therapies for overcoming the lethal disease.
